# Is There Any Reason to Prefer Cord Blood Instead of Adult Donors for Hematopoietic Stem Cell Transplants?

**DOI:** 10.3389/fmed.2015.00095

**Published:** 2016-01-11

**Authors:** Meral Beksac

**Affiliations:** ^1^Ankara University, Ankara, Turkey

**Keywords:** stem cell transplantation, engraftment, donor matching

## Abstract

As cord blood (CB) enables rapid access and tolerance to HLA mismatches, a number of unrelated CB transplants have reached 30,000. Such transplant activity has been the result of international accreditation programs maintaining highly qualified cord blood units (CBUs) reaching more than 600,000 CBUs stored worldwide. Efforts to increase stem cell content or engraftment rate of the graft by *ex vivo* expansion, modulation by molecules such as fucose, prostaglandin E2 derivative, complement CD26 inhibitors, or CXCR4/CXCL12 axis have been able to accelerate engraftment speed and rate. Furthermore, introduction of reduced intensity conditioning protocols, better HLA matching, and recognition of the importance of HLA-C have improved CB transplants success by decreasing transplant-related mortality. CB progenitor/stem cell content has been compared with adult stem cells revealing higher long-term repopulating capacity compared to bone marrow–mesenchymal stromal cells and lesser oncogenic potential than progenitor-induced stem cells. This chapter summarizes the advantages and disadvantages of CB compared to adult stem cells within the context of stem cell biology and transplantation.

Among many sources of hematopoietic stem cell sources, cord blood (CB) is the one that belongs to the earliest stages of life. Thus, CB has many features resembling fetal or even embryonic stem cells. This review will focus on biological features of CB and comparison of transplants with CB vs. adult stem cells.

## Cord Blood Storage Worldwide

More than three million units of CB are currently stored worldwide for autologous or altruistic purposes. Most recent bone marrow donors worldwide (BMDW) website shows that there are 676,446 unrelated CB units (CBUs) stored in 43 registries. This number is continuously increasing. Although CBUs belong to 2.5% of the stem cell pool, thanks to introduction of dCB transplants (dCBT) that contribute to approximately 20% of the unrelated stem cell transplants (1, 2). The usual odds ratio is 1 out of 100 CBUs that meet donor–recipient eligibility/match criteria and are transplanted. For a long time, low stem cell content was the major limiting factor against more frequent usage. The previous experience among pediatric patients have now expanded to adults, and the number of CB transplants has reached 30,000. Allogeneic unrelated CB transplants dominate the annual CBT statistics.

Cord blood units, unlike adult donor stem cell collection/harvests, are readily available on demand. Confirmatory HLA Typing and organization of transportation are the few barriers prior to CBT. Hence, CB offers an advantage for patients in need of urgent transplantation. As CBTs can be performed with a 4–6/6 match, this presents a chance of transplantation for patients who possess rare HLA types, i.e., who belong to ethnical groups not well-represented in registries or who belong to different race admixture families.

The HLA data of CBUs in the registries have improved in accordance with the required level and extend of typing as for the unrelated adult donors. CB banks are encouraged to perform collection, transportation, processing, and storage according to the standards published and audited by international authorities (FACT/NETCORD or AABB). The main purpose is to maintain high quality of stem cell products. Post-thaw stem cell counts are now being considered as the most important determinant of a successful engraftment (3). Purtill et al. have recently shown that CBUs from accredited banks engraft better.

## Amendment of Matching Criteria for Cord Blood

The matching criteria for unrelated CBUs, until very recently, were based on low/intermediate HLA-A,-B, and high-resolution HLA-DRB1 with a minimum match level of ≥4/6. In 2011, the EBMT and CIBMTR have published the importance of HLA-C typing. Compared to 8/8 matches, those mismatched at HLA-C had a higher transplant-related mortality (TRM) (HR: 3.97, *p* = 0.018). A 5/6 mismatch plus a HLA-C mismatch also increased the TRM (HR: 1.7, *p* = 0.029) (4).

Until recently, with the exception of high-resolution HLA-DRB1 typing, CBUs were typed at low-resolution level for HLA-A and -B loci. Recently, Oran et al. (5) have shown better high-resolution HLA-A-, HLA-B-, HLA-C-, and -DRB1-matched cords to determine the dominating CBU following double CB transplants. Additionally, they did not observe any 2-year transplant-related mortality among 7–8/8-matched patients. With increasing number of mismatches, TRM increased as well. Hence, better matching at a four digit level and four loci increases the success rate.

Although it may seem controversial, the Minnesota group recently published allele level mismatching based on the predominant unit to be only important for prevention of relapse (HR: 3.4, *p* = 0.01) (6). Otherwise, allele level mismatching was not associated with any significant detrimental effect on TRM, engraftment, or GVHD. In this study, there were only 32 out of 342 patients who were 9–10/10 allele matched. Although there was a reduction in TRM, it was not significant. One additional explanation for the difference between these two analyses could be due to comparison being performed at five or four loci. The Minnesota group proposes to aim for higher cell content units to overcome the potential problems that will arise from HLA mismatches. They argue a more selective approach trying to match CBUs at allelic level will decrease the number of CBTs. However, the New York Group has shown that by using a model in which both a higher eight-allele HLA match and a cell dose ≥2.0 × 10^7^/kg/unit were required, graft selection changed in 33% of transplants with minimal effect on cell dose (8.3% reduction). They conclude that while units chosen based on HLA-A, -B antigen, and -DRB1 allele match have substantial mismatch at higher resolution, and CB selection based on high-resolution HLA match is possible in a significant proportion of patients without compromising cell dose (7, 8).

The acceptability of HLA mismatches within the CBT setting can also be explained by matching for non-inherited parental HLA alleles (9). During pregnancy, fetal lymphocytes may be exposed to maternal antigens (NIMA) and gain tolerance. The maternal haplotype, which is not inherited can only be recognized by maternal HLA typing. NIMA matching has been shown to decrease graft-vs.-host disease (GVHD). CB Registries have now initiated the inclusion of maternal HLA types. In the case of a NIMA match, the 4/6 match can increase to a virtual 5/6 match grade. As a result, Europdonor and National Cord Blood program have shown that the elucidation of donors’ maternal HLA phenotypes can provide significant numbers of 6/6 and 5/6 virtually matched CBUs to patients and is potentially cost-effective (10).

In conclusion, recent data favor HLA-C, NIMA, and high-resolution typing to influence outcome and thus are recommended to be introduced into donor selection criteria. Although the role of HLA-C has been ascertained extensively, the impact of high-resolution typing and of which locus/loci remains to be investigated (6).

The number of HSCs transplanted in a CBT protocol is at least one log less than a PBSCT or BMT. On the other hand, these cells are cryopreserved and post-thaw viability may range between 75 and 99%. The outcome of CBT is affected negatively if recipient has developed anti-erythrocyte antibodies or allo-immunization prior to transplantation. In such situations, to avoid engraftment failure, the best HLA match and highest CD34 dose are required. However, the New York Group has published data negating the effects of allo-immunization (7, 8). Although an insignificant delay in median number of days for neutrophil engraftment (23 vs. 31 days) was observed, they have hypothesized that using an immunosupressive conditioning, lack of ATG and double unit grafts abrogates the deleterious effects of anti-HLA antibodies.

## Modification of Double Cord Blood Transplant Protocols

To increase immunosupression as a tool to overcome HLA mismatch effects, either fludarabine or ATG has been added to the conventional myeloablative conditioning (MAC) regimens. As summarized by Ruggeri et al. in the Eurocord/EBMT study, two MAC regimens were equally effective (11). The Fludara, Busulphan, and Thiotepa regimen (single CBT) or Fludara, Cyclophosphamide, and TBI regimens (dCBT) were the MAC protocols available widely. The Minnesota Group was the first to introduce non-myeloablative approach in CBT (12). The New York Group modified this regimen using cyclophosphamide (50 mg/kg), fludarabine (150 mg/m^2^), thiotepa (10 mg/kg), and 400 cGy total body irradiation with cyclosporine-A/mycophenolate mofetil immunosuppression without ATG. As a result they were able to achieve engraftment at a median of 26 days among 97% (13). Platelets recovered among 93% by day 180. Grades II–IV acute GVHD incidence was 67% at day 180, and chronic GVHD was 10% at 1 year. TRM was 20% at day 180, and relapse was 11% at 2 years. Overall, 2-year disease-free survival (DFS) was 60% at 2 years. Likewise, Kai et al. have reported results with a myeloablative regimen of CyTBI or CyTBI plus high-dose of cytosine arabinoside (14). This study once again proved the importance of HLA matching on engraftment. Furthermore, standard risk patients obtained a plateau of 67% event-free survival at 3 years compared to 29% among advanced disease patients (*p* = 0.023). In conclusion, introduction of NMA or RIC regimens have improved engraftment and EFS (40 vs. 60%) (6, 13).

## Cord Blood as a Source of Stem Cells

Umblical arterial CB vessels transport blood from the fetal hepatic circulation to the placental feto-maternal interface to be replaced with clean fresh blood devoid of metabolites. Umblical venous blood contains fetal cells, which have been in contact with placental intervillous syncytothrophoblasts. Oxygen and nutrient transport is maintained at this anatomical border throughout pregnancy. Placenta is an organ where cells of the fetus come into very close proximity but do not meet directly with maternal cells. The structure of placenta allows contact of fetal syncytothrophoblasts with maternal blood cells or decidua cells called interstitial trophoblasts. Successful pregnancy is maintained with suppression of immune reactivity either by lack of surface HLA expression, secretion of IGF-1/2, LIF, or TGF. This highly immunosuppressive environment contributes to the metabolomic profile of CB.

For many years, CB has been used for hematopoietic stem cell transplantation only. Recently, CB mesenchymal stromal cells (MSCs) have become clinically available. There is an estimated frequency of 1,000–5,000 MSCs in a typical UCB unit of approximately 100 mL (15). The main advantage of using CB-MSC is to enhance engraftment and to prevent or to treat GVHD. MSCs can be obtained from multiple sources, i.e., bone marrow, adipose or dental tissues, CB or Wharton’s jelly and even menstrual blood.

## Comparison of MSC from Different Sources

Comparison of MSCs obtained from different sources has been a topic of interest for many years. The decline in MSC number and differentiation potential with increasing age is a disadvantage against adult tissues, such as BM or adipose tissues. A comparison by Kern et al. was able to find no significant differences concerning the morphology and immune phenotype of the MSCs derived from different sources. However, differences could be observed concerning the success rate of isolating MSCs, which was 100% for BM and AT, but only 63% for UCB. The colony frequency was lowest in UCB, whereas it was highest in AT. However, UCB–MSCs possessed the longest culture period and showed the highest proliferation capacity, BM-MSCs possessed the shortest culture period and the lowest proliferation capacity. In a more recent study by Trivanović et al., in addition to immuno-phenotyping, proliferation and differentiation gene expression assays were also performed. Peripheral blood MSCs (PB-MSCs) and UC-MSCs showed the highest, while periodontal ligament MSCs (PDL-MSCs) and adipose tissue MSCs (AT-MSCs) the lowest values of both the replication potential and Relative Telomere Length. Although MSCs from exfoliated deciduous teeth (SHEDs), PDL-MSCs, and AT-MSCs showed higher mRNA expression of pluripotency markers, all MSCs expressed pluripotency marker proteins (16).

These two studies have shown that UC-MSCs have the highest proliferation rate and transplantation capacity than other MSCs (16, 17). If the origin is Wharton Jelly, MSCs are cultured efficiently because these cells are maintained in an early embryologic phase and therefore have retained some of the primitive stemness property resulting with a high proliferative capacity (18).

## Comparison of CB VS. IPS

One argument against storage of CB has arisen following successful generation of induced progenitor cells (IPSC). As these IPSCs have features similar to embryonic stem cells and can be obtained from adult tissues, they can be used in the same individual in need and from whom they were obtained. Currently, there are major obstacles against their routine use in clinical transplantation: their safety has not yet been proven and the production is very expensive and limited to advanced sites only.

Recently, a study comparing the MSC from CB vs. IPSC has shown that the CB MSC’s expression level of pluripotent genes OCT4 and Sox-2, Nanog and lin28 were significantly higher than those in IPSCs. In contrast, the expression level of oncogenic factors c-Myc and Klf4 were significantly higher in IPSCs than in CB-MSC. In conclusion, the authors suggested that CB-MSC is may be less oncogenic than IPSC. (19)

## Beneficial Immunological Features of Cord Blood

The typical feature of CB lymphocytes is as follows: the majority consists of antigen-inexperienced naïve lymphocytes, which are less responsive to allo-stimulation with a low effector cytokine production capacity. Furthermore, regulatory T cells (T regs) are seen more frequently. UCB dendritic cells have a lower antigen presenting activity. All these features are protective against GVHD but may predispose to graft failure or delay in immune reconstitution (20, 21).

On the other hand, UCB is rich in primitive CD16^−^CD56^++^ NK cells, which possess impressive proliferative and cytotoxic capacities and can be induced to expand using IL-12 or IL-15, so as to mount a substantial graft-vs.-leukemia (GvL) effect. The composition of CB mononuclear cells by Tregs and primitive NK cells is an ideal combination serving the purpose of GVL in the setting of allogeneic stem cell transplantation. This topic has been extensively addressed in a chapter in this issue of the journal.

## *Ex Vivo* Expansion and Engraftment Enhancers

Unlike all the factors addressed above, methods of CB expansion and engraftment include the most innovative approaches in the field of CBT and CB banking. They are described in detail in the current issue and elsewhere (22, 23, Beksac and Yurdakul, under review for publication in the same Research Topic).

In conclusion, this ready to use stem cell product has many clinically proven indications and protocols. More recent protocols for matching and transplantation have proven to improve transplant success. Furthermore methods under pre- and clinical investigations are very promising toward even better outcomes. As one of the pioneers in CBT has said this is a great time for those involved in CB biology and transplantation.

## Author Contributions

The author has defined and written the contents of the Research Topic and Mini Review.

## Conflict of Interest Statement

The author declares that the research was conducted in the absence of any commercial or financial relationships that could be construed as a potential conflict of interest.
